# Possible Mechanisms Underlying the Effects of Glucagon-Like Peptide-1 Receptor Agonist on Cocaine Use Disorder

**DOI:** 10.3389/fphar.2022.819470

**Published:** 2022-03-01

**Authors:** Changliang Zhu, Hailiang Li, Xuerui Kong, Yezhong Wang, Tao Sun, Feng Wang

**Affiliations:** ^1^ Department of Neurosurgery, General Hospital of Ningxia Medical University, Yinchuan, China; ^2^ Ningxia Key Laboratory of Cerebrocranial Disease, Yinchuan, China; ^3^ Incubation Base of National Key Laboratory, Ningxia Medical University, Yinchuan, China; ^4^ Department of Neurosurgery, The Second Affiliated Hospital of Guangzhou Medical University, Guangzhou, China; ^5^ Department of Neurosurgery, The First Affiliated Hospital of Zhejiang University School of Medicine, Hangzhou, China

**Keywords:** cocaine, glucagon-like peptide-1 receptor agonist, dopamine, glutamate, GABA, arachidonic acid, neuroinflammation

## Abstract

Cocaine use disorder (CUD) is a major public health challenge with a high relapse rate and lack of effective pharmacotherapies; therefore, there is a substantial need to identify novel medications to treat this epidemic. Since the advent of glucagon-like peptide-1 (GLP-1) receptors (GLP-1Rs) agonists (GLP-1RAs), their potential has been extensively explored and expanded. In this review, we first summarized the biological effects of GLP-1, GLP-1Rs, and GLP-1RAs. Subsequently, the recent literature examining the behavioral effects and the possible pharmacological mechanisms of GLP-1RAs on CUD was reviewed. Increasing preclinical evidence suggests that GLP-1RAs are promising in regulating dopamine release, dopamine transporter (DAT) surface expression and function, mesolimbic reward system and GABAergic neurons, and maladaptive behaviors in animal models of self-administration and conditioned place preference. In addition, the emerging role of GLP-1RAs in inhibiting inflammatory cytokines was reported. These findings indicate that GLP-1RAs perform essential functions in the modulation of cocaine-seeking and cocaine-taking behaviors likely through multifaceted mechanisms. Although the current preclinical evidence provides convincing evidence to support GLP-1RA as a promising pharmacotherapy for CUD, other questions concerning clinical availability, impact and specific mechanisms remain to be addressed in further studies.

## Introduction

Cocaine is an extraordinarily addictive drug extracted from the Erythroxylon coca plant which was originally used to relieve local pain (Biondich and Joslin, 2016). Cocaine is administered mainly through nasal, oral, and intravenous routes. Although cocaine was banned due to its addictive properties, at the beginning of the twenty century, it gained considerable popularity among young people worldwide. Indeed, cocaine is considered the most used illegal drug, with approximately 5.4% of adults having used it in Europe (MCDDA, 2019), and up to 20% of them becoming addicts (Wagner and Anthony, 2002; Reboussin and Anthony, 2006). According to the United Nations Office on Drugs and Crime, cocaine affected about 19 million people worldwide in 2018 (United Nations publication, 2020). Unfortunately, epidemiological surveys suggest that the number of cocaine consumers will continue to rise in the upcoming years (Hughes et al., 2016).

Cocaine use disorder (CUD) is generally believed to be an intractable brain disorder (O’Brien, 1997). The term CUD refers to various behaviors induced by repeated use of cocaine, ranging from sporadic or recreational use to abuse, dependence or addiction. Addiction is known as the most serious stage of CUD, which consists of physical and mental dependence. The former refers to physiological changes in the body, whereas the latter refers to specific mental effects on the central nervous system after repeated exposure to abused drugs. According to the incentive-sensitization addiction theory, individuals initially enjoy the euphoria caused by addictive drugs, then they gradually become addicted and develop tolerance to these drugs after repeated consumption (Berridge and Robinson, 1998). Subsequently, individuals completely lose self-control over the compulsive behaviors despite their strong willingness to discontinue drug consumption (Berridge and Robinson, 1998). As a result, once the drug use is discontinued, individuals experience a range of serious withdrawal symptoms, culminating in a craving-induced intractable relapse, which is considered a great challenge in clinical treatment (Hunt et al., 1971; O’Brien, 1997).

CUD continues to be a major health issue characterized by a high relapse rate, which causes several catastrophic outcomes for addicts as well as their families and the society, such as low productivity, high rate of divorce or crimes, the spread of AIDS or hepatitis, and even suicide (Padmanathan et al., 2019; World Health Organization, 2019; Strang et al., 2020). Unfortunately, there are currently no Food and Drug Administration (FDA)-approved medications despite decades of studies aimed at searching for effective pharmacotherapies for this disease (Pierce et al., 2012). In addition to the wide variety and widespread availability of psychostimulants, another reason for disastrous consequences may be related to the few efficacious treatment options available after becoming addicted to these drugs. Therefore, there is a clear need to identify new therapeutic drugs to combat this epidemic. However, the development of therapeutic drugs appears to be progressing slowly despite extensive efforts to reduce these devastating outcomes. One of the possible explanations is the incomplete understanding of the neurobiological mechanisms of psychostimulants, resulting in the low efficacy of available therapeutic drugs in achieving persistent abstinence and stopping addiction-like behaviors. Existing evidence indicates that there is a tremendous need for additional studies that focus on new neurobiological mechanisms of cocaine and novel strategies in treating CUD (Kampman, 2019; Volkow, 2020).

In recent years, there has been considerable progress in the field of drug addiction research wherein the potential of glucagon-like peptide-1 (GLP-1) receptors (GLP-1Rs) were elucidated. The pharmacological actions of GLP-1RAs has been widely explored and expanded. For instance, preclinical literature shows that GLP-1RAs have important impacts on the regulation of dopamine release as well as on the mesolimbic reward system and the amelioration of cocaine-mediated behavior in animal models of CUD (Hernandez and Schmidt, 2019). However, the precise mechanisms of their pharmacologic actions remain elusive.

In this review, a brief overview of the biological actions of GLP-1, GLP-1R and GLP-1RAs is presented. Furthermore, we outlined the behavioral effects of GLP-1RAs and their potential mechanisms. Understanding the underlying mechanisms of the GLP-1 system on reward-related behavior will pave the way for the development of effective pharmacological interventions for CUD. Finally, future studies and therapeutic implications are proposed.

## Biological Actions of GLP-1s, GLP-1Rs and GLP-1RAs

Glucagon-like peptide-1(GLP-1) is a 30-amino acid anorexigenic neuropeptide produced that is largely both in the small intestine and in the nucleus tractus solitarius (NTS) of the caudal brainstem that stimulates insulin secretion, reduces glucagon secretion, decelerates gastric emptying and suppresses overall food intake after a meal (Holst, 2007; Reddy et al., 2014). GLP-1 possesses a short half-life because it can be rapidly inactivated by the dipeptidyl peptidase-4 (DPP4), which is highly expressed throughout the brain (Crews et al., 2017). Given these biological properties, GLP-1 synthetic compounds, which are resistant to DPP4 degradation and possess longer half-life, are already utilized for the clinical treatment of type 2 diabetes (Pi-Sunyer et al., 2015; Drucker et al., 2017).

The biological effects of GLP-1 depend greatly on the activation of GLP-1Rs. The GLP-1R is a G-protein coupled receptor that is abundantly distributed throughout the brain including the mesolimbic reward system such as the ventral tegmental area (VTA), accumbens core (NAc) and lateral septum (LS) (Hernandez and Schmidt, 2019). GLP-1Rs are also found outside the mesolimbic reward system including the hippocampus (Risold and Swanson, 1996; Lathe, 2001; Davidson et al., 2007) and laterodorsal tegmental nucleus (Hernandez et al., 2020), which are involved in the behaviors of cocaine-seeking and cocaine-taking in rodents (Hernandez and Schmidt, 2019). Previous studies suggested that the activation of GLP-1Rs can enhance intracellular calcium concentration and activate protein kinase A (PKA), protein kinase C (PKC), and phospholipase C (PLC) (Hayes et al., 2011a). Additionally, the activation of GLP-1Rs is reported to effectively suppress the reinforcing properties of cocaine in an animal model with intravenous cocaine self-administration (Schmidt et al., 2016). Importantly, it is also implicated in an increase in the frequency of action potential firing in medium spiny neurons (MSNs) and the prevention of the cocaine-seeking behavior (Hernandez and Schmidt, 2019) in an animal model of human relapse. Consistent with these findings, previous research also observed that animals with a GLP-1R knockout displayed increased locomotor activity and conditioned place preference (CPP), signifying the enhanced reinforcement effects of cocaine in these animals (Harasta et al., 2015). Furthermore, renewed attention is focused on GLP-1Rs due to their promising action on regulating GABAergic circuits, dopamine release and cocaine-induced behavior in cocaine-seeking animal models (Hernandez et al., 2020). Consequently, GLP-1Rs may represent novel molecular targets for the treatment of CUD.

In general, GLP-1Rs can be activated mainly through GLP-1RAs (Hernandez et al., 2020). To date, many available GLP-1RAs (e.g., lixisenatide, liraglutide, exenatide, dulaglutide, and semaglutide) readily penetrate the blood-brain barrier (Holscher, 2010) and have been widely approved for the clinical treatment of type II diabetes by the FDA (Gentilella et al., 2019). Administration routes of GLP-1RAs have become more convenient with the advent of the oral administration of semaglutide. Newer agonists that have different pharmacokinetic and pharmacodynamic actions have longer half-life and display more beneficial actions on glycaemic control and body weight reduction than GLP-1. The FDA-approved GLP-1RAs are based on the exendin-4 or GLP-1 structure. Exendin-4 is a GLP-1RA originally extracted from the saliva of the lizards (Holst, 2007). Additionally, exendin-4 is one of the most widely used GLP-1RAs and its therapeutic effects have been extensively explored in animal models of CUD.

## Effects of GLP-1 and GLP-1RA on Food Intake

Previous studies have demonstrated that peripheral and central homeostatic modulators of body weight interact with and affect the mesolimbic reward system (Kenny, 2011; Williams and Elmquist 2012; Grill and Hayes, 2012). Furthermore, metabolic factors, such as GLP-1, inhibit VTA dopamine neurons and food intake (Gutzwiller et al., 1999a; Gutzwiller et al., 1999b; Reddy et al., 2014; Volkow et al., 2017). Similarly, GLP-1RAs have also attracted considerable interest due to their possible benefits in reducing the consumption of palatable food (Williams and Elmquist 2012; Mietlicki-Baase et al., 2014) and treating many neurodegenerative diseases characterized by dopamine alterations (Bassil et al., 2014). Importantly, food intake and substance use disorder share a common neurobiological mechanism to some extent (Kenny, 2011). Based on their ability to modulate the mesolimbic reward system and food intake, it could be hypothesized that GLP-1 and GLP-1RAs may regulate drug-motivated behaviors. Indeed, GLP-1 and GLP-1RAs received growing appreciation due to their involvement in the drug-reward mechanism (Hernandez and Schmidt, 2019; Eren-Yazicioglu et al., 2021). In addition, GLP-1 is believed to be implicated in the behavioral improvement of laboratory rodents exposed to many psychostimulants such as cocaine, alcohol, nicotine, and amphetamine (Egecioglu et al., 2013a; Graham et al., 2013; Sorensen et al., 2015; Tuesta et al., 2017; Hernandez and Schmidt, 2019). For example, GLP-1 signaling showed promising efficacy in reducing reward-seeking behavior and intake of nicotine in animal models with intravenous nicotine self-administration (Tuesta et al., 2017). Collectively, GLP-1 and GLP-1RAs deserve more attention in the management of addiction and future addiction research.

## Effects of GLP-1RA on the Preclinical Models of Cocaine Use Disorder

One of the major contributions in an important emerging area over the past decades has progressed towards understanding the pharmacological actions of GLP-1RAs, which are traditionally approved for the treatment of type 2 diabetes and obesity (Drucker et al., 2017). The emerging ability of the GLP-1RA exendin-4 to ameliorate the rewarding and reinforcing behaviors triggered by cocaine is well summarized in previous literature (Hernandez and Schmidt, 2019). The first study investigating the capacity of GLP-1RAs for mediating the behavioral responses of addictive drugs demonstrated that exendin-4 was adequate to reduce reward-related behavior induced by drugs in mice. Systemic pretreatment with exendin-4 (2.4 μg/kg) dose-dependently inhibited the amphetamine and cocaine-induced behavior including the acquisition and expression of conditioned place preference (CPP) (Egecioglu et al., 2013b). A similar behavioral impact of exendin-4 on blunting cocaine-mediated CPP response was observed in another study determining that cocaine-related CPP was significantly reduced by peripheral pretreatment with exendin-4 (10.0, 30.0 and 100.0 μg/kg, i.p.) (Graham et al., 2013). Consistent with these two studies, systemic administration of exendin-4 was effective in inhibiting nicotine-mediated CPP and reducing the release of extracellular dopamine (Egecioglu et al., 2013a). Collectively, these studies comprehensively explored the suppressing effects of the systemic administration of exendin-4 on drug-induced CPP in an animal model to examine the rewarding properties of psychostimulant drugs (Tzschentke, 1998; Bardo and Bevins, 2000). Consistent with these results, our previous work also showed that systemic administration of exendin-4 dose-dependently modulated the cocaine-induced behavior such as acquisition and expression of CPP, and the extinction and reinstatement of cocaine-conditioned behavior (Zhu et al., 2021). Overall, this evidence suggests that the systemic administration of GLP-1RAs is effective in weakening the rewarding properties of injectable cocaine. However, it may be arbitrary to draw comprehensive conclusions from these promising findings that GLP-1RA exendin-4 directly attenuated the pleasurable and rewarding properties of cocaine. Moreover, exendin-4 (0.25 μg/kg) was shown to produce nausea/malaise (Kanoski et al., 2011). Additionally, 0.3 μg/kg of exendin-4 could effectively reduce locomotion in rodents (Sørensen et al., 2015). Consequently, the suppressive effects of exendin-4 on cocaine CPP in freely-moving mice may be either due to either nausea or malaise or locomotor activity inhibition rather than specific effects on drug reward.

In addition to the CPP paradigm, the potential role of exendin-4 in another preclinical paradigm of self-administration, which was considered the most popular and important approach for examining drug-seeking, was also well-investigated. Previous studies showed an essential and underappreciated efficacy of exendin-4 on animal model of cocaine self-administration. For example, both systemically and centrally administered exendin-4 in animal cocaine self-administration models improved cue-and cocaine-triggered reinstatement (Sørensen et al., 2015; Schmidt et al., 2016) and amphetamine-induced locomotion (Erreger et al., 2012). Consistent with these results, active lever response measurement in animals pretreated with fluoro-exendin-4 (3.0 μg/kg) revealed that peripheral administration of exendin-4 significantly inhibited cocaine-seeking behavior with modest negative effects such as pica food intake reduction, and body weight loss (Hayes et al., 2011b; Kanoski et al., 2011) that were also suspected of influencing cocaine-seeking behavior. Indeed, previous studies demonstrated that GLP-1RAs are effective in blocking the reinstatement of cocaine-seeking behavior, because these doses have been shown to induce unexpected effects such as nausea, malaise, and inhibition of locomotor activity (Sørensen et al., 2015). To explore the dose of systemic administration of exendin-4 that prevented cocaine-seeking behavior without these negative effects, a study conducted by Hernandez and his colleagues found that peripheral fluoro-exendin-4 (0.01, 0.1, and 0.2 μg/kg) did not only avoid the abovementioned negative effects but also significantly blocked the reinstatement of drug-seeking behavior evoked by cocaine (Hayes et al., 2011a). Moreover, the systemic administration of the GLP-1RA fluoro-Ex-4 (0.2 μg/kg) revealed an easy penetration of the blood-brain barrier and selectively decreased cocaine-seeking behavior without producing adverse malaise-like symptoms (Hernandez et al., 2018). The preclinical study investigating the underappreciated role of GLP-1RAs indicates that direct infusions of exendin-4 (0.005 or 0.025 μg) into the LDTg before the commencement of the reinstatement test dose-dependently blocked cocaine-seeking behavior but did not lead to body weight reduction in animals (Hernandez, et al., 2020). Overall, these studies show that exendin-4 selectively attenuates cocaine-seeking behaviors without producing malaise-like symptoms. Therefore, future research should utilize behaviorally selective doses of exendin-4 that suppress cocaine-taking-and cocaine-seeking behaviors.

Taken together, numerous studies have demonstrated a promising role of behaviorally relevant doses of GLP-1RAs in the regulation of maladaptive behaviors in preclinical models, but there is still little information regarding the precise mechanisms underlying these behavioral effects of GLP-1RAs.

## Potential Mechanisms Underlying the Effects of GLP-1RAs on Cocaine Use Disorder

To date, there is currently no efficacious treatment for CUD due, at least in part, to an incomplete understanding of the neurobiological mechanisms of this mental disorder. Consequently, a comprehensive understanding of the potential mechanisms underlying CUD is necessary to identify new therapeutic drugs to combat this epidemic. A variety of significant advances in the pharmacological mechanisms of exendin-4 are summarized in this review. It is worth noting that instead of an independent mechanism, the mechanisms underlying the effects of GLP-1RAs on CUD may be multifaceted and complex.

### Mesolimbic Dopamine Mechanism

The mesolimbic dopamine system is commonly believed to be the most important and classical mechanism shared by all addictive substances (Nestler, 2005). This system was seen to play an essential role in the pathogenesis of many brain disorders characterized by aberrant levels of dopamine, such as Alzheimer’s disease, Huntington’s diseases, and Parkinson’s disease (Li et al., 2009; Li et al., 2010; Mitchell et al., 2011; Gardoni and Bellone, 2015). In 1954, Olds and Milner opened the door to probing the function of the mesolimbic reward system by self-administering electrical stimulation into specific brain regions of rodents. Subsequently, they successfully identified some neuronal circuits that were widely recognized as the mesolimbic dopamine system implicated in mediating the rewarding effects of addictive drugs (Olds and Milner, 1954). This system is composed of functionally and anatomically interrelated nuclei that function as modulators of the motivational, emotional, and other incentive behaviors (Pierce and Kumaresan, 2006; Schmidt and Pierce, 2010) that underlie the reinforcing behaviors of cocaine (Pierce and Kumaresan, 2006). Thus, identifying modulators of the mesolimbic dopamine system may lay the foundation for developing novel medications for the treatment of CUD (Volkow et al., 2017).

Cocaine can inhibit the reuptake of extracellular neurotransmitter dopamine at dopamine terminals by binding to and blocking the dopamine transporter (DAT) (Berridge and Robinson., 1998; Volkow and Wise, 2005), leading to a dramatic elevation in synaptic dopamine concentration. Dopamine is commonly considered an active agent that underlies the rewarding and reinforcing properties of addictive drugs (Volkow and Wise, 2005). Therefore, cocaine acts by affecting the mesolimbic reward region and finally leading to cocaine-intaking and cocaine-seeking behaviors. Consequently, some agents that enhance DAT function and attenuate dopamine bioavailability could be applied for the amelioration of cocaine-induced addiction-like behaviors. Consistent with this hypothesis, accumbal microdialysis measurements in drugs-experienced mice showed that systemic pretreatment with exendin-4 (2.4 μg/kg, i.p.) blocked dopamine elevation (Egecioglu et al., 2013b). Furthermore, systemic exendin-4 (30.0 μg/kg, i.p.) reduced locomotor hyperactivity and cocaine-mediated dopamine elevation in the striatum (Sørensen et al., 2015). In line with these data, a recent study indicated that intra-lateral ventricular exendin-4 also blocked cocaine-mediated dopamine secretion using microdialysis in freely moving mice (Fortin and Roitman, 2017). Notably, previous research also showed that GLP-1 positively regulates the surface expression and affects the function of DAT (Reddy et al., 2014; Reddy et al., 2016). Furthermore, microdialysis experiments in freely moving mice demonstrated that infusions of exendin-4 (2.4 μg/kg, i.p.) significantly diminished cocaine-triggered dopamine levels and indirectly affected DAT expression and function in the lateral septum (Reddy et al., 2016). Collectively, it is conceivable that the inhibitory effects of peripheral GLP-1RAs on addiction-like behavior induced by cocaine are supposed to be associated with the activation of DAT inhibition and reduction in extracellular dopamine. One limitation of the above studies, however, is that they completely fail to explore the direct brain region of exendin-4’s action. Indeed, systemic administration of exendin-4 is distributed throughout the whole brain (Hernandez and Schmidt, 2019). Accordingly, it is conceivable that the pharmacological actions of exendin-4 on cocaine-mediated behaviors may be strongly related to other brain regions other than the mesolimbic dopamine systems. Consequently, it is not easy to elucidate the specific brain areas that may mediate the functional significance of exendin-4.

Regardless of the direct targets of exendin-4, these findings suggest that the GLP-1RA exendin-4 could be repurposed to attenuate cocaine-related behaviors, partly through affecting DAT function and suppressing dopamine signaling. In addition, these findings indicate an important target for the development of treatments for CUD by regulating dopamine signaling. However, *one fly in the ointment* of these current studies is that the exact mechanisms underlying the beneficial impacts of exendin-4 on dopamine and maladaptive behaviors have yet to be fully untangled.

### Glutamate Mechanism

In addition to the mesolimbic dopamine system, another important mechanism is related to glutamate which is commonly referred to as the major excitatory neurotransmitter (Siegel et al., 2006). Accumulating evidence suggests that repeated exposure to cocaine can induce persistent neuroadaptation of glutamatergic signaling in the nucleus accumbens, contributing to addiction-related changes such as neuronal dysfunction, neurotoxicity enhancement, cognitive impairment, and cocaine-induced behavior promotion (Kalivas and Nakamura, 1999; Schmidt and Pierce, 2010). Indeed, a series of adaptational changes in glutamate release, re-uptake, receptor expression, and intracellular signaling following repeated exposure to cocaine was observed in NAc (Schmidt and Pierce, 2010). Overall, these studies indicate that the persistent alterations in glutamate transmission may mediate alterations in behaviors after drug exposure. Consistent with these data, previous studies indicate that long-term adaptative changes in glutamate signaling were most likely involved in drug-related behavior (Jones and Bonci, 2005). Additionally, glutamate receptors including NMDA and AMPA receptors reportedly played a role in the maladaptive behaviors of addiction (Kelley, 2004; Engblom et al., 2008). Importantly, exendin-4 was shown to reduce AMPA receptor expression and mediate excitatory postsynaptic currents (EPSCs) in VTA dopamine neurons that project to the nucleus accumbens shell (Engblom et al., 2008; Wang et al., 2015). Furthermore, AMPA/NMDA EPSC ratio in the VTA was also decreased following exendin-4 administration (Wang et al., 2015). Altogether, these results indicate that GLP-1RAs may play an essential role in the transmission of excitatory and inhibitory neurotransmitters. Additionally, similar mechanisms (i.e., weakening excitatory strength and/or enhancing inhibitory inputs) may underlie the promising effects of GLP-1RAs on cocaine reward.

### γ-Aminobutyric Acid Mechanism

γ-Aminobutyric acid (GABA) is sensed by GABA_A_ and GABA_B_ receptors. The former is implicated in the mediation of synaptic and tonic currents, while the latter is a G protein coupled receptor traditionally known as an inhibitory neurotransmitter receptor in the central nervous system (Bowery et al., 2002). Additionally, GABA_B_ is closely associated with CUD. For example, the GABA_B_ agonist baclofen was shown to attenuate cocaine-mediated self-administration in laboratory rats (Cousins et al., 2002). Hence, GABA_B_ plays a potential pivotal role in CUD, and activating GABA_B_ may be an effective strategy in treating this disease. Of interest, the GLP-1RA liraglutide is validated to be effective in mediating GABA signaling (Babateen et al., 2017). GLP-1R is abundantly expressed in the GABA neurons in the laterodorsal tegmental nucleus (LDTg) (Hernandez et al., 2018) and activation of GLP-1Rs can inhibit cocaine-seeking behaviors (Hernandez et al., 2020). Moreover, they found that GLP-1R can be activated by exendin-4 application (Hernandez et al., 2020). GLP-1RAs could effectively inhibit cocaine-seeking behavior by activating the LDTg GABA neurons. Consequently, LDTg GABA neurons may potentially be the target of GLP-1RA exendin-4’s pharmacological action in cocaine (Hernandez et al., 2020). Consistent with these results, the patch-clamp recordings found that GLP-1 and exendin-4 temporarily regulated synaptic and tonic currents activated by GABA in CA3 pyramidal neurons, and thereby augmenting GABA signaling in CA3 Pyramidal Neurons (Jin et al., 2015; Babateen et al., 2017; Korol et al., 2015). Overall, the translational implications of these findings indicate an essential yet largely unexplored effects of GLP-1RAs on LDTg GABA neurons and a novel neurobiological mechanism by which GLP-1RAs attenuate cocaine-seeking and reinstatement. Thus, these emerging studies further expand the potential effects of GLP-1RAs on CUD from distinct perspective, highlighting the significance of the innovative research focused on the neurocircuits and neurobiological mechanisms underlying the pharmacological actions of GLP-1RAs. Nevertheless, caution is necessary because few studies have investigated the functional associations between GABAergic LDTg signaling and motivational behaviors of cocaine to date.

Taken together, although emerging evidence suggests that the suppressing effects of GLP-1RAs on cocaine-mediated behaviors may be associated with GABA signaling, more studies are required to uncover the detailed mechanisms by which systemic GLP-1RAs modulate GABAergic LDTg signaling and other relevant neural activity. For example, exendin-4 activates presynaptic GLP-1Rs and regulates GABAergic neuronal activity via glutamatergic mechainsms (Mietlicki-Baase et al., 2014), which may explain the role of exendin-4 in cocaine-induced behavior amelioration from a different perspective. Therefore, these findings may represent an important step in the conceptually innovative approaches in searching for an effective pharmacotherapy for CUD.

### Arachidonic Acid Mechanism

Recent evidence on the exploration of the pharmacological mechanism of exendin-4’s actions indicates that in addition to the classical dopaminergic, glutamatergic and GABAergic pathways, the arachidonic acid (AA) pathway is also implicated in CUD. AA is thought to be involved in enhancing pro-inflammatory cytokines in the brain (Farooqui et al., 2007). Psychostimulants are seen to raise AA levels and may exacerbate oxidative stress and addiction-like behavior (Kovacic, 2005; Poon et al., 2007; Ng et al., 2008). Based on the above findings, regulating expression of AA and influencing oxidative stress may open a novel door for suppressing psychostimulants actions. Importantly, a recent study found that GLP-1RA can medicate cocaine’s actions by targeting AA (Reddy et al., 2016). In this study, the application of exendin-4 not only abolished the cocaine-mediated increase of DA but also enhanced the surface expression and function of DAT and induced a reduction in the expression of the 2 arachidonylglycerol (2-AG) and AA after repeated cocaine exposure (Reddy et al., 2016). These results altogether suggest that GLP-1RA is effective in regulating DAT function and expression and enhancing dopamine reuptake, and thereby leading to a reduction in the cocaine-mediated dopamine level and inhibition of dopamine-associated behavior (Reddy et al., 2016). These pharmacological actions are attributable, at least in part, to blocking the expression of the retrograde messengers including endocannabinoid 2- arachidonylglycerol (2-AG) and arachidonic acid in the lateral septum (Reddy et al., 2016). Consistent with these results, previous study suggested that diminished levels of AA after cannabinoid CB1 receptor antagonist administration can affect the function of the dopamine transporter, thereby suppressing cocaine-induced reward-seeking behaviors (Xi et al., 2008). Altogether, these findings indicate that GLP-1RAs may be effective in promoting dopamine re-uptake by reducing AA expression, which may inhibit the cocaine-triggered increase in dopamine concentration and cocaine-related behaviors. However, caution is essential when interpreting mechanisms from this study. Indeed, the exact role of decreased AA and 2-AG levels in addiction-like behaviors has not been examined to date. Additionally, how GLP-1RA attenuates the expression of 2-AG and AA and regulates neural activity has not been investigated.

### Neuroimmune Mechanisms

One of the pronounced advancements in the past is a substantial expansion of knowledge concerning the role of neuroinflammation in CUD. Another progress is the demonstration of the relationship between neuroimmune and GLP-1RAs (Chaudhuri et al., 2012). For instance, growing evidence suggested that both neuroimmune system (Clark et al., 2013; Lacagnina et al., 2017) and GLP-1RAs (Hernandez and Schmidt, 2019) have significant effects on CUD.

The neuroinflammatory pathway is triggered by pattern recognition receptors (PRR), including pathogen or danger-associated patterns (PAMPs and DAMPs) which address pathogen invasion or tissue injury. Activation of the toll-like receptor 4 (TLR4), considered the most common PRR, enhances MyD88 activation, resulting in the increase of nuclear factor-κB (NF-κβ) and important inflammatory cytokine including IL-1β and TNF-α (Brown et al., 2011). IL-1β is produced by the cleavage of caspase-1, the inactive precursor of IL-1β, which addresses specific immune receptor activation including the TLR4/IL-1R1 inflammasome complex (Mangan et al., 2018.). TNF-α is one of several known cytokines released in response to ligands binding to TLR4 (Bohannon et al., 2013). Intriguingly, previous evidence suggested that cocaine docks to the domain of MD-2 of TLR4 and enhances TLR4/IL-1β signaling that was believed to be involved in regulating dopamine levels and reward-related behaviors such as cocaine-induced conditioned place preference and self-administration after repeated exposure to cocaine (Northcutt et al., 2015). Congruent with these findings, studies in animals demonstrated that TLR4 may have an essential and underappreciated role in reward-related behaviors induced by alcohol (June et al., 2015), morphine (Hutchinson et al., 2012), and cocaine (Kashima and Grueter, 2017). In addition, the TLR3 and TLR2 signaling pathways were shown to increase cocaine consumption (Blednov et al., 2017; Zhu et al., 2018; Blednov et al., 2020). Furthermore, cocaine alone is sufficient to upregulate the expression of other cytokines including NF-κβ (Zhu et al., 2021), TNF-α (Lewitus et al., 2016; Brown et al., 2018), IL-1β (Northcutt et al., 2015), and IL-8 (Meckel and Kiraly, 2019), which in turn enhances dopamine release and finally exaggerates the drugs-mediated behavioral response. Moreover, MyD88 signaling was demonstrated to be effective in the modulation of addiction-like behaviors (Rivera et al., 2018). Therefore, TLR-related signaling pathways may provide a fresh perspective for the development of novel therapies for CUD.

Indeed, many anti-inflammatory ingredients that can block cocaine-associated neuroinflammation in the brain perform an important function in alcohol abuse treatment (Kohno et al., 2019). For example, n-acetylcysteine, an antioxidant and anti-inflammatory agent provide effective treatment for several psychiatric and neurological disorders characterized by dysfunction in oxidative stress, neuroinflammation, as well as glutamate and dopamine signaling (Deepmala et al., 2015). Agreeing with these data, the specific TLR4 antagonist naltrexone plays an important role in the dopamine increase and development of cocaine-induced place preference in rodents (Northcutt et al., 2015). Indeed, a range of anti-inflammatory agents with an ability to decrease expression of IL-1β and TNF-α, such as Ibudilast (Ray et al., 2014) and Minocycline (Garrido-Mesa et al., 2013) exhibit promising actions on treatments for substance use disorders. Importantly, the anti-inflammatory cytokines IL-10 effectively modulates anxiety-like behaviors after repeated exposure to substances (Patel et al., 2021) and reduces alcohol intake by mediating GABA signaling (Russell et al., 2021). Collectively, anti-inflammatory agents with the ability to weaken the TLR4-related neuroinflammation may be repurposed to reduce the release of extracellular neurotransmitters and thereby treat CUD.

Intriguingly, GLP-1RAs have received widespread attention in recent years due to their neuroprotective (Holst et al., 2011; Zhu et al., 2021) and anti-inflammatory effects (Hattori et al., 2010; Chaudhuri et al., 2012; Lee and Jun 2016). For example, exendin-4 has been reported to be effective in reducing inflammation indices, including TLR2, TLR4, NF-κβ, tumor necrosis factor (TNF)-α, and interleukin (IL)-1β (Chaudhuri et al., 2012; Kozela et al., 2017; Li et al., 2020), which have been heavily implicated in cocaine-related memories (Correia et al., 2020). Consequently, it is possible that exendin-4 influences drug-associated memories and ameliorate addiction-like behaviors by inhibiting neuroinflammation. Consistent with this hypothesis, there is growing evidence that regulation of cocaine-mediated enhancement of TLR4, NF-κβ, TNF-α and IL-1β signaling may block the rewarding properties of cocaine (Padhye et al., 2009; Albensi and Mattson, 2015; Lewitus et al., 2016; Northcutt et al., 2015; Kozela et al., 2017; Brown et al., 2018; Zhu et al., 2021). In addition, anti-inflammatory agents, such as naltrexone, cannabidiol, garcinol, ibudilast and minocycline, have also been shown to play a role in drugs addiction through direct and indirect inhibition of TLR4 or the downstream cytokine activation (Monsey et al., 2017; Dunbar and Taylor, 2017; Kohno et al., 2019; Northcutt et al., 2015). These findings indicate that neuroinflammatory markers may function as a bridge between GLP-1RAs and addiction-associated behaviors and represent an important avenue for developing novel strategies to reduce cocaine relapse. Additionally, GLP-1RAs can break neuroinflammatory bridges and influence neurotransmitter modulation including that of dopamine, glutamate, GABA, and even cause addiction-like behaviors. However, caution is warranted when interpreting pharmacological mechanisms of the GLP-1RAs from the above studies. Indeed, the role of TNF-α in cocaine-mediated memory remains controversial to date (Correia et al., 2020). Additionally, little is known about the mechanisms by which GLP-1RAs exert these actions. Consequently, further research should focus on the exploration of possible associations among CUD, neuroinflammation, and GLP-1RAs.

## Conclusion

There is growing enthusiasm for GLP-1RAs due to their emerging role in many chronic brain disorders, including CUD. The evidence outlined herein indicates that GLP-1RAs may represent an important step in the development of novel drug therapies for CUD. Accordingly, previous studies proposed that metabolic compounds including GLP-1RAs should be viewed as promising modulators of addiction-like behavior (Engel and Jerlhag, 2014; Jerlhag, 2018). However, till date, the association between endogenous levels of metabolic agents and CUD in humans remains unclear. For instance, n-acetylcysteine reduced cocaine intake and locomotor sensitization as well as reinstatement in animal models but had no effect on cocaine intake in humans (Madayag et al., 2007). Consistent with this data, the recent human study also demonstrated that exenatide diminished levels of GLP-1 and insulin but failed to show inhibitory effects on cocaine-associated behaviors (Angarita et al., 2021), indicating that more clinical studies are needed to investigate the effects of GLP-1 on patients with CUD. In addition to regulating the release of neurotransmitters including dopamine, GABA, and glutamate, GLP-1RAs may have an important role in cocaine reward by reducing inflammation. These studies indicate that both cocaine exposure and type II diabetes may affect an individual brain’s physical function through a common mechanism, and that anti-diabetic drugs, such as GLP-1RAs can be repurposed to treat CUD. However, the detailed mechanisms about how GLP-1 signaling functions to regulate reward-related behavior remain unknown. Consequently, further investigations are necessary to understand the cellular, molecular and neurophysiological mechanisms underlying the behavioral effects of GLP-1 signaling. Advancement in our understanding of the behavioral benefits of GLP-1 signaling on cocaine-induced behavior is conducive to identifying novel synthetic ligands with higher affinity, thereby providing information for the development of pharmacotherapies for CUD. The renewed interest in GLP-1 signaling represents an essential step in the continued elaboration of the potential effects of central signaling involved in maladaptive behaviors of cocaine. In addition, clinical studies have shown that women tend to use cocaine at an earlier age, although men are more likely to abuse cocaine (Weiss et al., 1997a; Weiss et al., 1997b); there are more girls than boys aged 12 to 18 involved in cocaine use (Anker et al., 2007). Furthermore, central estrogen signaling is reported to play a key role in the actions of GLP-1 on reward-related behavior (Richard et al., 2016). Consequently, it is feasible to speculate that the role of GLP-1RAs may incorporate sex-specific effects in animal models of CUD but lack sufficient evidence. However, few studies investigated the influence of gonadal hormones on the ability of GLP-1RAs to block cocaine-mediated incentive behaviors to date. This review will hopefully inspire more research on the role of GLP-1 signaling in the rewarding and reinforcing properties of cocaine and other addictive drugs. Nevertheless, a key open question in preclinical studies is that these promising results are currently derived from rodents and lack relevant evidence from patients addicted to cocaine. In the field of addiction research, whether the effect of oral GLP-1RAs displays the similar effects as injectible GLP-1RAs on drug addiction remains to be demonstrated. This information is relevant as more oral GLP-1RAs are approved for clinical application. Furthermore, future clinical experiments are essential in bringing to fruition the promising results from preclinical studies, and thereby identifying effective medications for the treatment of this intractable brain condition characterized by recurrent relapse.
